# Determinants of Length of Stay Following Total Anterior Circulatory Stroke

**DOI:** 10.3390/geriatrics2030026

**Published:** 2017-08-04

**Authors:** James P. Curtain, Man Yu, Allan B. Clark, Nicholas D. Gollop, Joao H. Bettencourt-Silva, Anthony Kneale Metcalf, Kristian M. Bowles, Marcus D. Flather, John F. Potter, Phyo Kyaw Myint

**Affiliations:** 1 Ageing Clinical & Experimental Research Team, Institute of Applied Health Sciences, School of Medicine, Medical Sciences & Nutrition, University of Aberdeen, Aberdeen AB25 2ZD, UK; 2 Norwich Medical School, University of East Anglia, Norwich NR4 7TJ, UK; yu_0912@hotmail.com (M.Y.); Allan.Clark@uea.ac.uk (A.B.C.); n.gollop@doctors.org.uk (N.D.G.); kristian.bowles@nnuh.nhs.uk (K.M.B.); m.flather@uea.ac.uk (M.D.F.); John.Potter@uea.ac.uk (J.F.P.); phyo.myint@abdn.ac.uk (P.K.M.); 3 Clinical Informatics, Department of Medicine, University of Cambridge, Cambridge CB2 1TN, UK; jhb56@medschl.cam.ac.uk; 4 Stroke Research Group, Stroke Services, Norfolk and Norwich University Hospital, Norwich NR4 7UY, UK; kneale.metcalf@nnuh.nhs.uk

**Keywords:** haemorrhagic stroke, ischaemic stroke, length of stay, mortality, total anterior circulatory stroke

## Abstract

Identification of factors that determine length of stay (LOS) in total anterior circulatory stroke (TACS) has potential for targeted intervention to reduce the associated health care burden. This study aimed to determine which factors predict LOS following either ischaemic or haemorrhagic TACS. The study sample population was drawn from the Norfolk and Norwich Stroke and Transient Ischemic Attack (TIA) Register (1996–2012), a prospective registry. 2965 patients admitted with TACS verified by a stroke specialist team were included. Primary analysis identified predictors of length of stay (LOS) in either haemorrhagic or ischaemic TACS. Secondary analyses identified predictors of LOS in patients who were discharged alive or who died during admission separately. Moderate (*p* = 0.014) to severe disability (*p* = 0.015) and history of congestive heart failure (*p* = 0.027) in the primary analysis and pre-stroke residence in a care facility among patients who survived to discharge (*p* = 0.013) were associated with a shorter length of stay. Factors associated with increased length of stay included presence of neurological lateralisation in the primary analysis (*p* = 0.004) and amongst patients who died (*p* = 0.003 and *p* = 0.014 for ischaemic and haemorrhagic stroke, respectively). Patients with advanced age (≥85 years) with haemorrhagic stroke had longer LOS regardless of mortality outcome. Patients with low pre-morbid disability (modified Rankin score ≤2 who died following haemorrhagic TACS also had longer LOS. Our study found predictors of LOS following TACS include neurological lateralisation, pre-stroke disability status, congestive heart failure, pre-morbid residence and age. The identification of such factors would assist in resource allocation and discharge planning.

## 1. Introduction

Stroke is a leading cause of death and disability worldwide with an annual incidence of 152,000 per year in the UK [1] and 795,000 in the United States [2]. The cost of providing acute and long-term care, along with estimated loss of productivity costs in stroke patients, is over 8 billion pounds per year [3] in the UK. Annual costs in the United States reach 33.6 billion dollars, of which 7.6 billion dollars are attributed directly to inpatient stays [2] representing a major economic burden to the health services. 

Length of stay (LOS) in acute medical care is a significant contributor to the financial implication of caring for stroke patients and the ability to accurately predict which patients are likely to require longer inpatient care would be desirable for both budgetary planning and healthcare provider considerations, and also in communicating with patients and families to manage expectations at a vulnerable and uncertain time [4]. 

Total anterior circulation stroke (TACS), as defined by the Oxford Community Stroke Project classification [5], constitutes around 17% of stroke [5] and is a severe form of stroke associated with higher mortality rates [6], higher healthcare costs [7] and longer length of stay [8] than other stroke subtypes. Deeper insight and better understanding of factors that determine LOS in TACS creates the potential for targeted intervention to reduce the associated health care burden.

We [9,10] and others [11] have derived and validated clinical prediction tools to predict length of stay in acute stroke, but mostly in cohorts of stroke patients with varying severity. We recently identified major determinants of mortality in TACS and derived a novel scoring system, the TACS-6 score, to predict mortality [12]. However, there is no tool in current clinical use that has been derived to specifically predict length of stay following this most severe form of stroke. The purpose of this study was to identify what factors in TACS patients were associated with hospital length of stay.

## 2. Methods

The study participants were drawn from the Norfolk and Norwich Stroke and Transient Ischemic Attack (TIA) Prospective Register that has routinely collected data from its inception (Nov 1996) at the Norfolk and Norwich University Hospital. The patients included in the study were admitted between 1996 and 2012. The hospital is a regional tertiary care hospital with a catchment population of ~750,000, which provides a comprehensive stroke care service including a hyper acute stroke unit, a step-down acute stroke unit and multi-disciplinary medical and rehabilitation care. Data collection methods were previously reported [13,14]. 

For the purposes of this study, patients diagnosed with a TACS and entered into the registry for the first time were included. TACS was defined as a significant ischemic or hemorrhagic event affecting the middle and/or anterior cerebral artery territories. Clinically, a diagnosis of TACS was made by the presence of all three of the following: unilateral weakness (and/or sensory impairment) of face, arm and leg, homonymous hemianopia and higher cortical dysfunction (dysphasia and/or visuospatial disorder). If lateralisation (i.e., unilateral weakness) was absent, the diagnosis was made radiologically, such as by arterial territory involved and degree of the neurological deficit in conjunction with other clinical signs (e.g., eye deviation/obvious neglect). Computed tomography (CT) was the first line imaging modality, with Magnetic Resonance Imaging (MRI) performed in CT negative cases. Not all ischaemic stroke underwent CT angiography in addition to non-contrast CT.

Variables of interest included age, sex, stroke sub-type (ischemic or hemorrhagic), pre-stroke functional status as a marker of frailty assessed using the pre-stroke modified Rankin score (mRs 0 = no symptoms, mRs 5 = severe disability, completely dependent on care) and pre-stroke residence. Patients with modified rankin scores 4 and 5 (moderately severe and severe disability) were combined for the analysis as these represented collectively a vulnerable group. We considered sheltered housing as a pre-care home state (*N* = 8) and mental health facility (*N* = 2) as a care setting. Comorbidities were retrieved from electronic discharge letters on the patient administration system. Comorbidities (including atrial fibrillation (AF), diabetes mellitus, myocardial infarction (MI), chronic kidney disease, and congestive heart failure) that could influence LOS were included. Comorbidities with less than 5 patients recorded were excluded from the analyses as comparisons with these groups would not be statistically useful. Length of stay was considered as the period in days from date of admission to date of discharge from the hospital for those who survived or from date of admission to date of death for those patients who died as an inpatient. 

Descriptive statistics for each potential explanatory factor was given as the number and percentage in each category. A linear regression model was used to identify the association between potential explanatory factors and length of stay. When there was less than 5 events the category it was excluded from the model. The assumptions standard regression analysis was not met as the residuals were not normally distributed, hence a non-parametric bootstrap was used to estimate the p-values and confidence intervals. 1000 iterations of the bootstrap were used. Statistical significance was considered as *p* ≤ 0.05. Statistical analysis was performed using Stata 14 (StataCorp, College Station, TX, USA).

Ethical approval was obtained from the Newcastle and Tyneside National Health Service (NHS) Research Ethics Committee (12/NE/0170) and the study protocol was approved by the Steering Committee of the Register. 

Given the inherent differences in management and prognosis, the analyses were conducted for ischemic and hemorrhagic stroke specifically in the whole sample. Subgroup analysis was then performed to examine the factors associated with length of stay separately for patients who survived to discharge and those who died as inpatients in line with our previous work [15].

## 3. Results

A total of 2965 patients with total anterior circulation stroke (42.0% male, mean age 76.9 ± 10.8 years, 58.0% female, mean age 81.5 ± 9.3 years) were included in the current study. The median age of the whole sample was 81 years (range = 20–102 years; IQR 74–86 years). There were 232 (7.8%) aged less than 65 years, 1717 (57.9%) aged 65–84 years and 1016 (34.3%) aged 85 years or more. 2439 (82.2%) had an ischemic stroke. 526 (17.7%) had a haemorrhagic stroke. 2693 (90.8%) patients were admitted with clinical features consistent with lateralisation of neurological deficit. 456 (15.4%) patients were admitted from a care home. 1477 (58.7%) were fully independent prior to admission with baseline modified Rankin scores of 0. Data for modified Rankin scores was available for 2517 out of 2965 patients. 486 (16.4%) had atrial fibrillation, 290 (9.8%) had congestive heart failure, 54 (1.8%) had chronic kidney disease, 150 (5.1%) had a previous myocardial infarction and 88 (3%) were diabetic. 1707 patients (57.6%) died during their admission, of whom 1379 (46.5%) patients had an ischaemic TACS and 328 (11.1%) patients had a haemorrhagic TACS. Table 1 shows the sample characteristics.

Table 2 details the length of stay analysis separately for the ischemic and hemorrhagic stroke subtypes. In the ischemic stroke group, moderate (mean LOS 16 days, *p* = 0.014) and moderately severe or severe disability (mean LOS 15 days, *p* = 0.015) prior to TACS (modified Rankin score 3 and 4 or 5) were both significantly associated with shorter length of stay as compared with those who had no baseline disability (modified Rankin score 0). In the haemorrhagic stroke group, the presence of lateralisation (mean LOS 16 days, *p* = 0.004) was associated with a longer length of stay and those patients with a history of congestive heart failure (mean LOS 5 days, *p* = 0.027) had significantly shorter length of stay.

Table 3 and Table 4 show the stroke subtype specific length of stay results for the patients who were alive at discharge or who died as inpatients respectively. In patients who survived an ischemic TACS, those who lived in a care home prior to admission had significantly shorter length of stay (mean LOS 20 days, *p* = 0.013). In those who survived a bleed, patients with advanced age (mean LOS 33 days for patients aged ≥85 years, *p* = 0.006) had significantly longer LOS. In patients who died following an ischemic event, the presence of lateralisation (mean LOS 12 days, *p* = 0.003) was associated with increased length of stay. Amongst patients who died following a haemorrhagic TACS, the presence of lateralisation (mean LOS 10 days, *p* = 0.014), advanced age (mean LOS 11 days for patients aged ≥85 years, *p* = 0.04) and modified Rankin scores 1 (mean LOS 15 days, *p* = 0.042), 2 (mean LOS 19 days, *p* = 0.025) as compared with those who had a baseline score of 0 were associated with longer of lengths of stay.

## 4. Discussion

We have identified several factors associated with both shorter and longer length of stay in patients who presented with a total anterior circulation stroke. Predictive criteria such as the PLOS score [11] includes variables such as stroke severity assessed by NIHSS score, stroke type, decreased consciousness on admission, congestive heart failure and atrial fibrillation to predict length of stay but its generalizability has been questioned [16] and it was not specific to TACS patients, who often suffer the greatest burden of post-stroke disability and the most prolonged length of stay. 

To date, much of the literature has focussed on stroke severity, rather than on specific clinical features of neurological deficit, as a predictor of length of stay, with increasing stroke severity scores associated with longer inpatient admission [17,18]. However, lateralization forms part of the criteria of assessment in several validated stroke severity scoring tools, including the NIHSS score and the Canadian Neurological Score [19,20,21]. Early functional improvement in paretic limbs following anterior circulation stroke has been shown to predict greater recovery while those limbs lacking voluntary movement in the few days following the occurrence of stroke were associated with poorer outcome [22]. Our study had similar findings with lateralisation increasing length of stay in those patients who died of ischaemic or haemorrhagic TACS and increasing overall inpatient stay in haemorrhagic TACS, with such a deficit likely reflecting increased severity of stroke and vulnerability to complications of immobility, including infection, venous thrombo-embolism and pressure sores. Severity scoring tools were not included in the analysis for the current study. Rather, the inclusion of a broader binary measure of impairment (lateralization or not) instead of the specific grade of limb weakness may have decreased the potential for inter-rater variability in the assessment of neurological deficit.

Advancing age has previously been shown to be predictive of longer length of stay [23] and poor outcomes in stroke, including change in residence to nursing home care reflecting an individual’s increased dependence and care needs if they survived the stroke [24]. In clinical practice the delayed availability of such care often represents a rate-limiting aspect to speed of discharge in health care systems such as in the National Health Service in the UK. The current finding of advanced age being associated with increased length of stay is supported by a recently validated length of stay prediction tool [10] with a similar cohort mean age being observed in both studies, and differs to earlier studies which had younger cohorts that did not show an association between age and length of stay [18]. An ever-increasing elderly population is a prominent factor in contemporary medical practice that places further demands on health services. Recognising the impact age has on length of stay has therefore important resource allocation implications, emphasising the requirement for early review of patient’s pre & post-morbid functional status, detailed collateral history at the point of admission and a multi-disciplinary (including social worker) review given the higher likelihood that an elderly patient will need greater resource (i.e., care or change in residency to nursing home) utilisation. The earlier thus work could be done for these elderly patients the more efficiently their LOS could be influenced. Equally, stroke services should take into account patient demographics such as age, not just the number of population they serve, so stroke unit beds are able to cope with increasing ageing populations.

Congestive heart failure (CHF) has previously been reported to prolong length of stay following stroke [11,25] and transient ischaemic attack [26], forming one of the criteria in the aforementioned PLOS score. In keeping with prolonged length of stay CHF has been reported as a predictor of increased cost following stroke [25,27]. It could be expected that increased comorbidity prior to TACS would contribute to more prolonged recovery, placing further limitation on rehabilitation effort and exacerbating vulnerability to complications that accompanies an acute insult such as a TACS. However, in the current study patients with a haemorrhagic stroke and a history of CHF had approximately only one third of the mean length of stay of those patients who were without pre-morbid cardiac impairment. CHF has also been associated with increased mortality following stroke [28] and this may also account for the decreased length of stay in the haemorrhagic TACS cohort in the current study, where patients with poor physiological reserve succumbed having suffered the most severe form cerebrovascular injury. It would be of interest to observe similar findings in future studies to establish this link further. Cardiac failure and cerebrovascular disease share similar risk factors, such as hypertension, atrial fibrillation and hypercholesterolaemia (as a contributor to ischaemic cardiomyopathy). The association of poor outcomes between heart failure and stroke underlines the importance of risk factor modification and achieving primary and secondary prevention targets.

Higher pre-stroke modified Rankin scores have previously been reported to influence length of stay and mortality [29]. In the primary analysis a reduced length of stay was seen in patients with higher disability (mRs group 3 and 4 or 5) who suffered an ischaemic TACS. This may in part be due to higher inpatient mortality rates being seen in the study’s cohort who had a pre-stroke mRs of 3 (*p* = 0.003) and 4 (*p* = 0.014) and an associated relatively shorter LOS. Equally, among those who survived to discharge, patients who had higher degrees of disability may have been less likely to benefit from intensive, acute stroke rehabilitation prompting more timely discharge back to their pre-stroke residence with appropriate care arrangements that were already catering for their needs. This is also reflected by the fact patients admitted from care settings had shorter LOS. Amongst those patients who died, minimal to mild pre-morbid disability predicted increased length of stay following a haemorrhagic event. This longer length of stay likely reflects that these patients were now significantly unwell following their event but may have had some degree of physiological reserve that enabled them to survive for a period of time before succumbing to their acute illness. Equally, discharge planning may have been a factor with these patients becoming more care dependent following such an event and their prolonged stay reflecting the time requirement to arrange new, suitable discharge destinations such as a high intensity nursing home or hospice.

The main study limitation is that it is based on one centre’s experience in the UK NHS setting and patient cohort where stroke management resources, delivery and patient characteristics may differ in other places and settings. Also, the analysis was conducted retrospectively. However, the data were collected prospectively, except for co-morbidities, which were documented prospectively in the medical notes and identified at a later stage using electronic records. This prospective data collection of consecutive admissions and the lack of exclusion criteria to the register limit the possibility of selection bias and reflect a real world picture. 

## 5. Conclusions

In summary, the identification of contributors to length of stay would aid the development of useful prediction criteria in future studies. This study with a large cohort of patients contributes valuable information to predicting which patients will have an extended or likely shorter inpatient stay following a TACS. This information can potentially be used for planning of services and projections of future health care costs in stroke populations with knowledge about the incidence of TACS within local stroke patient populations. Further studies in multiple settings would be beneficial to validate predictive criteria for TACS length of stay given its wide-ranging impact on healthcare providers and patients.

## Figures and Tables

**Table 1:** Total anterior circulation stroke patient cohort characteristics.

	Total	%	Ischemic	%	Hemorrhagic	%
**Stroke Type**						
Ischaemic	2439	82.3	-		-	
Haemorrhage	526	17.7	-		-	
**Mortality**						
Survived	1258	42.4	1060	43.5	198	37.6
Died	1707	57.6	1379	56.5	328	62.4
**Age**						
<65 years	232	7.8	167	6.9	65	12.3
65–85 years	1717	57.9	1381	56.6	336	63.9
≥85 years	1016	34.3	891	36.5	125	23.8
**Gender**						
Female	1719	58.0	1452	59.5	267	50.8
Male	1246	42.0	987	40.5	259	49.2
**Lateralization**						
Yes	2693	90.8	2256	92.5	437	83.1
No	272	9.2	183	7.5	89	16.9
**Residence**						
Care Home	456	15.4	399	16.3	57	10.8
Home Alone	988	33.3	829	34.0	159	30.2
Home Family	1521	51.3	1211	49.7	310	59.0
**Rankin Score**						
mRs ^1^ 0	1477	58.7	1175	56.8	302	67.46
mRs 1	206	8.2	175	8.4	31	6.9
mRs 2	235	9.3	193	9.3	42	9.4
mRs 3	312	12.4	264	12.8	48	10.7
mRs 4 or 5	287	11.4	262	12.7	25	5.6
**Comorbidity**						
AF ^2^ No	2479	83.6	1965	80.6	514	97.7
AF Yes	486	16.4	474	19.4	12	2.3
CHF ^3^ No	2675	90.2	2155	88.4	520	98.9
CHF Yes	290	9.8	284	11.6	6	1.1
CKD ^4^ No	2911	98.2	2385	97.8	526	100
CKD Yes	54	1.8	54	2.2	0	0
MI ^5^ No	2815	94.9	2312	94.8	503	95.6
MI Yes	150	5.1	127	5.2	23	4.4
DM ^6^ No	2877	97.0	2352	96.4	525	99.8
DM Yes	88	3.0	87	3.6	1	0.2

^1^ mRs = modified Rankin scale; ^2^ AF = atrial fibrillation; ^3^ CHF = congestive heart failure; ^4^ CKD = chronic kidney disease; ^5^ MI = myocardial infarction; ^6^ DM = diabetes mellitus.

**Table 2:** Adjusted combined length of stay analysis with 95% confidence intervals of ischaemic and haemorrhagic TACS.

	Ischaemic TACS					Haemorrhagic TACS				
	N	Mean	SD	95% CI	*p*-Value	N	Mean	SD	95% CI	*p*-Value
**Age**										
<65 years	167	19.7	23.5			65	13.8	17.9		
65–84 years	1381	17.8	17.7	−5.87–2.08	0.350	336	15.1	17.4	−5.98–4.17	0.727
≥85 years	891	16.9	18.1	−6.64–1.92	0.280	125	16.2	18.9	−5.42–6.88	0.816
**Gender**										
Female	1452	17.7	18.3			267	15.2	17.6		
Male	987	17.6	18.3	−2.34–1.15	0.506	259	15.1	18.1	−2.07–4.81	0.436
**Lateralisation**										
No	183	15.2	21.1			89	9.7	18.3		
Yes	2256	17.8	18	−2.07–5.01	0.415	437	16.3	17.5	2.32–11.9	0.004
**Residence**										
Home Alone	829	18.5	20			159	16.2	19.2		
Home Family	1211	17.9	18.1	−3.05–0.88	0.280	310	15	17.2	−5.67–2.81	0.509
Care home	399	15.1	14.5	−4.77–0.74	0.152	57	13.4	17.3	−9.74–4.03	0.416
**Rankin Score**										
mRs 0	1175	19.1	19.4			302	15	18.6		
mRs 1	175	18.8	20.3	−3.63–3.01	0.856	31	20	19.4	−1.72–13.7	0.128
mRs 2	193	18.4	20.4	−3.63–2.65	0.760	42	16.7	17.4	−3.74–7.69	0.498
mRs 3	264	16	15.3	−5.12–−0.5	0.014	48	16.8	17.5	−3.90–6.86	0.590
mRs 4 or 5	262	14.9	15.3	−6.15–−0.6	0.015	25	12.4	13.3	−7.37–5.41	0.763
**Comorbidity**										
AF No	1965	17.5	18			514	15.3	17.9		
AF Yes	474	18.2	19.4	−1.55–3.14	0.506	12	10.2	12.9	−9.62–9.56	0.996
CHF No	2155	17.5	17.7			520	15.3	17.9		
CHF Yes	284	18.7	22.5	−1.53–4.39	0.345	6	5.8	5.6	−18.4–−1.09	0.027
CKD No	2385	17.6	18.3			526	15.2	17.8		
CKD Yes	54	17	17.1	−4.73–5.56	0.875	0	-	-		
MI No	2312	17.5	17.9			503	15	17.4		
MI Yes	127	19.2	24.3	−3.78–6.08	0.647	23	18	25.4	−7.29–14.5	0.518
DM No	2352	17.7	18.4			525	15.2	17.8		
DM Yes	87	16.4	15.4	−2.49–5.17	0.492	1	1	-		

**Table 3:** Adjusted length of stay analysis with 95% confidence intervals of ischaemic and haemorrhagic TACS in patients who were alive at discharge.

	Ischaemic TACS					Haemorrhagic TACS				
	N	Mean	SD	95% CI	*p*-Value	N	Mean	SD	95% CI	*p*-Value
**Age**										
<65 years	119	25.1	25.5			40	19.6	20.4		
65–84 years	669	24.4	19.7	−6.71–3.64	0.561	129	24.1	21.7	−5.37–9.76	0.569
≥85 years	272	25.2	20.7	−6.75–5.37	0.823	29	33.3	25.1	5.15–30.5	0.006
**Gender**										
Female	612	24.9	21			102	25.4	22.7		
Male	448	24.3	20.2	−3.41–2.36	0.720	96	23.6	21.8	−5.21–8.87	0.611
**Lateralisation**										
No	59	29.3	29.1			23	19.9	30.8		
Yes	1001	24.4	20.1	−16.2–0.84	0.077	175	25.2	20.9	−7.79–22.2	0.346
**Residence**										
Home Alone	346	26.6	23.3			66	26.6	24.8		
Home Family	591	24.4	19.8	−6.08–0.82	0.135	114	23.4	20.1	−11.6–4.50	0.387
Care home	123	20.4	15.8	−12.2–−1.43	0.013	18	24.5	25.6	−20.4–10.1	0.506
**Rankin Score**										
mRs 0	625	25	21.7			125	24.4	23.3		
mRs 1	73	25.3	24.5	−5.70–5.99	0.962	10	30.1	22.2	−7.54–22.2	0.334
mRs 2	79	24.1	16.1	−5.10–3.30	0.674	17	17	14.8	−18.8–2.88	0.150
mRs 3	90	24.6	18.2	−4.59–4.43	0.973	14	30.6	24.7	−10.4–17.6	0.612
mRs 4 or 5	79	21.4	17.8	−7.13–4.89	0.715	6	22.3	21.8	−18.5–9.01	0.499
**Comorbidity**										
AF No	902	24.3	20.5			196	24.6	22.3		
AF Yes	158	27	21.5	−3.52–4.88	0.750	2	17.5	17.7		
CHF No	979	24.1	20.2			197	24.6	22.3		
CHF Yes	81	31.8	25	−0.11–11.1	0.055	1	16	-		
CKD No	1043	24.6	20.7			198	24.6	22.2		
CKD Yes	17	32.1	21.5	−1.85–17.2	0.114	0	NA	NA		
MI No	1017	24.3	20.5			193	24	21.4		
MI Yes	43	32.4	22.6	−0.88–14.3	0.083	5	45.2	43.3		
DM No	1033	24.8	20.7			198	24.6	22.2		
DM Yes	27	21	19.1	−10.6–7.29	0.720	0	NA	NA		

**Table 4:** Adjusted length of stay analysis with 95% confidence intervals of ischaemic and haemorrhagic TACS in patients who died during admission.

	Ischaemic TACS					Haemorrhagic TACS				
	N	Mean	SD	95% CI	*p*-Value	N	Mean	SD	95% CI	*p*-Value
**Age**										
<65 years	48	6.2	7.4			25	4.5	6.2		
65–84 years	712	11.7	12.8	2.82–8.59	0.000	207	9.4	10.8	−0.27–7.60	0.068
≥85 years	619	13.3	15.4	3.79–10.6	0.000	96	11	12.8	0.23–10.3	0.040
**Gender**										
Female	840	12.4	13.8			165	8.9	8.9		
Male	539	12	14.3	−1.56–2.11	0.768	163	10.1	13.2	−0.32–5.20	0.084
**Lateralisation**										
No	124	8.5	10.8			66	6.2	9.2		
Yes	1255	12.6	14.2	1.28–6.18	0.003	262	10.3	11.6	0.80–6.93	0.014
**Residence**										
Home Alone	483	12.7	14.6			93	8.8	8.2		
Home Family	620	11.6	13.8	−2.69–1.26	0.477	196	10	12.9	−1.60–4.67	0.337
Care home	254	13	12.8	−2.26–3.16	0.744	39	8.3	8	−7.65–2.36	0.300
**Rankin Score**										
mRs 0	550	12.3	13.5			177	8.4	10.1		
mRs 1	102	14.2	15.2	−2.02–4.60	0.445	21	15.1	16.4	0.28–14.5	0.042
mRs 2	114	14.5	22.1	−2.66–5.34	0.512	25	16.5	19.3	1.05–15.7	0.025
mRs 3	174	11.5	11.3	−4.01–0.48	0.123	34	11	8.9	−0.55–6.83	0.095
mRs 4 or 5	183	12.1	13.2	−4.39–1.07	0.233	19	9.3	7.9	−1.68–7.65	0.209
**Comorbidity**										
AF No	1063	11.7	13.1			318	9.5	11.2		
AF Yes	316	13.7	16.7	−1.19–3.87	0.298	10	8.7	12.5	−8.71–13.1	0.693
CHF No	1176	12	12.9			323	9.6	11.3		
CHF Yes	203	13.5	19.1	−1.61–4.60	0.344	5	3.8	2.8		
CKD No	1342	12.3	14.1			328	9.5	11.2		
CKD Yes	37	10.1	8.3	−7.08–0.12	0.058	0	NA	NA		
MI No	1295	12.2	13.3			310	9.4	11.3		
MI Yes	84	12.4	22.4	−6.24–5.32	0.877	18	10.4	10.7	−4.80–5.78	0.855
DM No	1319	12.1	14			327	9.5	11.2		
DM Yes	60	14.3	13.1	−1.55–6.27	0.237	1	1	NA		
